# Implementation of Electronic Adherence Monitors and Associated Interventions for Routine HIV Antiretroviral Therapy in Uganda: Promising Findings

**DOI:** 10.3389/fdgth.2022.899643

**Published:** 2022-07-22

**Authors:** Jessica E. Haberer, Robert Baijuka, John Bosco Tumuhairwe, Edna B. Tindimwebwa, James Tinkamanyire, Ellyk Tuhanamagyezi, Lawrence Musoke, Lindsey E. Garrison, Marisa DelSignore, Nicholas Musinguzi, Stephen Asiimwe

**Affiliations:** ^1^Center for Global Health, Massachusetts General Hospital, Boston, MA, United States; ^2^Department of Medicine, Harvard Medical School, Boston, MA, United States; ^3^Kabwohe Clinical Research Centre, Kabwohe, Uganda

**Keywords:** HIV, implementation science, electronic adherence monitoring, SMS intervention, Africa

## Abstract

**Background:**

High, sustained adherence is critical for achieving the individual and public health benefits of HIV antiretroviral therapy (ART). Electronic monitors provide detailed adherence information and can enable real-time interventions; however, their use to date has largely been confined to research. This pilot study (NCT03825952) sought to understand feasibility and acceptability a relatively low-cost version of this technology and associated interventions for routine ART delivery in sub-Saharan Africa.

**Methods:**

We provided two ART clinics in rural, southwestern Uganda with electronic adherence monitors for data-informed counseling as well as optional SMS messages to clients and/or social supporters (daily or triggered by missed or delayed doses) and/or an alarm. Clinic and ART client experiences were observed for 3 months per client, including time and motion studies. Qualitative interviews among clients, clinicians, and healthcare administrators were informed by the Consolidated Framework for Implementation Research.

**Results:**

Fifty-one ART clients were enrolled; 57% were male and the median age was 34 years. Choice of associated intervention varied among participants. The median number of visits during follow-up was two per client. Counselors reviewed the adherence data with 90% of clients at least once; 67% reviewed data at all visits. Average adherence was 94%; four clients had adherence gaps >1 week. Acceptability was high; all but one client found the monitor "very useful” and all found SMS “very useful.” Clinic visits among clients with the intervention lasted 4 min longer on average than those in standard care. The monitors and daily SMS generally functioned well, although excess SMS were triggered, primarily due to cellular network delays. Overall, participants felt the technology improved adherence, clinic experiences, and clinician-client relationships. Few worried about stigma and privacy. Cost was a concern for implementation, particularly at scale.

**Conclusion:**

We successfully implemented a relatively low-cost electronic ART adherence monitor and associated interventions for routine care in rural Uganda. Feasibility and acceptability were generally high, and individuals were identified who could benefit from adherence support. Future work should involve longitudinal follow-up of diverse populations, clinical outcomes, and detailed cost-effectiveness analysis to help drive policy decisions around the uptake of this technology for routine clinical care.

**Clinical Trial Registration:**

identifier: NCT03825952.

## Introduction

High and sustained adherence to HIV antiretroviral therapy (ART) is well-known to play a central role in achieving viral suppression, which is critical for individual health benefits as well as prevention of secondary viral transmission (1, 2). Adherence monitoring provides valuable information on the effectiveness of ART as well as enables targeted use of limited resources for adherence interventions. In routine clinical care, adherence is most commonly measured through self-report, if assessed at all. While simple, inexpensive, and quick, it often underestimates non-adherence due to social desirability and recall bias (3). Other alternatives include pill counts, pharmacy refill tracking, pharmacokinetic measures, and electronic monitors, each with their own strengths and weakness (4–6).

Electronic adherence monitoring involves the use of a “smart” pill box that records a time-and-date stamp each time it is opened as a proxy for medication ingestion. This data can either be stored on the monitor for later downloading when a patient comes to clinic, or it can be transmitted in real-time through cellular networks, depending on the technology used. While limited by potential inaccuracies (i.e., opening the monitor without dosing or removal of multiple pills at a time for later dosing), these monitors uniquely provide a day-to-day record of adherence behavior. This information can be used to identify specific periods of non-adherence, which can be useful for tailoring adherence counseling and/or delivery of “just-in-time” adherence intervention. For example, real-time monitors can automatically trigger text message reminders if a dose is not taken within a defined time period and/or connections can be made to known social support systems (7). Alternatively, incentives can be delivered for demonstrated adherence (8, 9). Electronic adherence monitors have been shown to be acceptable and improve ART adherence in multiple, although not all, contexts (7–12); however, their use has largely been limited to research studies because of expense and concerns about impact on clinical operations. Because the cost of these devices has been reduced dramatically in recent years, they now have potential for use in routine clinical care, and study of their acceptability and impact on routine clinical experiences is warranted.

Implementation science aims to improve the uptake of evidence-based interventions through systematic assessment of potential barriers and facilitators (13). The Consolidated Framework for Implementation Research (CFIR) is helpful to understand the multiple constructs that can influence intervention adoption, namely the intervention itself (e.g., features, relative advantage, cost), the individual (e.g., knowledge, preferences), the inner setting (e.g., clinical structures, staffing, culture), the outer setting (e.g., healthcare policies and expectations), and the implementation process (e.g., rollout plans, leadership models) (14). We previously conducted a qualitative study with healthcare administrators and clinicians and ART clients that explored relevant factors for uptake of real-time electronic adherence monitors and associated interventions for routine HIV care in Uganda according to the CFIR (15). In brief, participants viewed the monitors and associated interventions favorably, reporting that they would be beneficial for supporting adherence and improving clinical outcomes. At the individual level, participants felt that a desire for good health and a welcomed pressure to adhere favored implementation of the technology, although some participants were worried that clients would not use the monitors as directed and that poverty, stigma, and privacy concerns might inhibit their use. Within the clinic setting, participants felt that the adherence data would likely improve the quality of counseling and thereby clinical staff morale, as well as increase the efficiency of care delivery (e.g., targeting counseling only for those who demonstrated adherence challenges). They emphasized the need for proper training on use of the technology. They also stated that community influences, international norms around the importance of supporting adherence, and availability of funding would be important outer setting considerations. Other relevant factors included existing infrastructure and care expenditures. Additionally, participants felt the implementation process would need to be guided by the Ministry of Health (MoH) and other funders with attention to sustainability, the appropriate target populations for its use, and coordination across the health care system.

Here, we present a pragmatic, follow-up study in which we used mixed methods to understand the experience of implementing relatively low-cost, real-time electronic adherence monitors and associated interventions in two clinics providing ART in rural southwestern Uganda. The first clinic has experience with research, whereas the other is prototypical of HIV care in the region. The objective was to understand the feasibility, acceptability, and practical implications of this technology with minimal support from our research team.

## Methods

### Intervention

**Figure 1:**
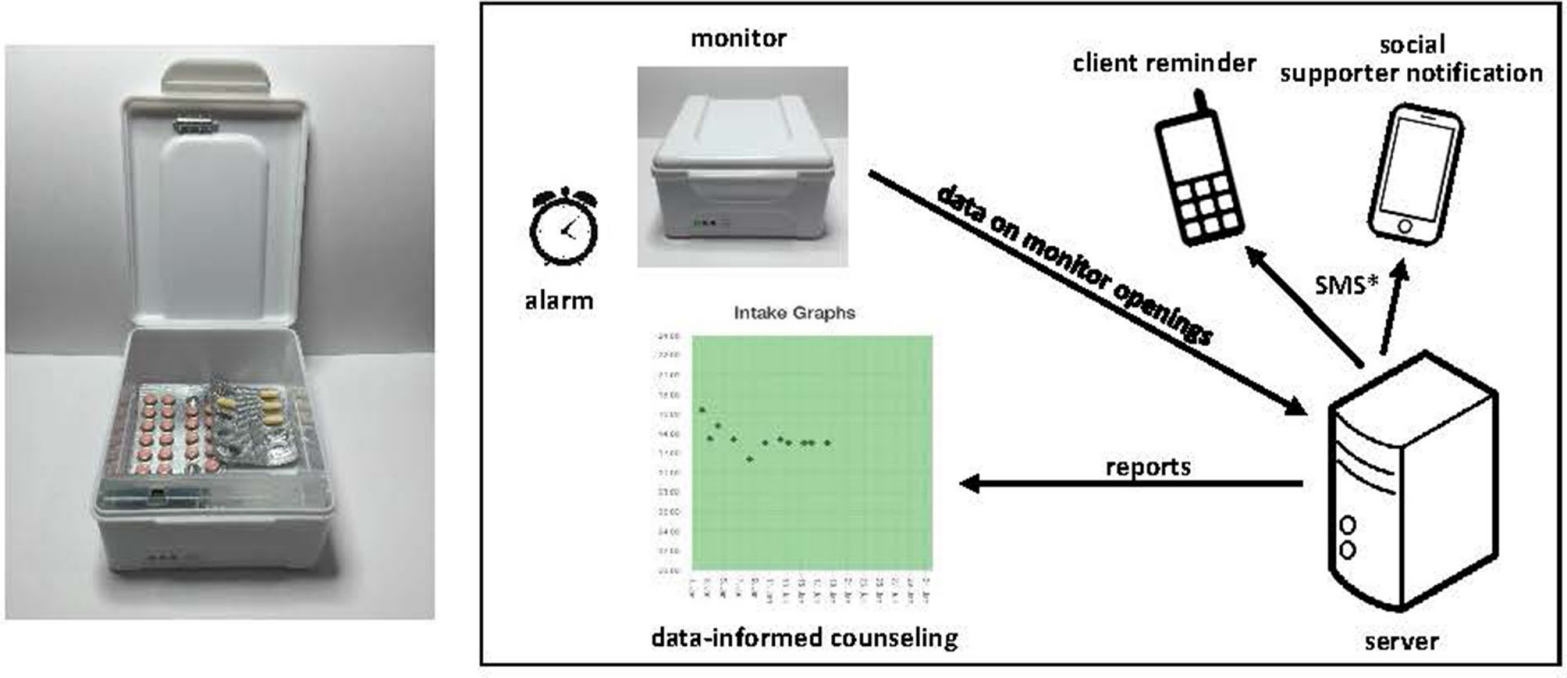
The evriMED monitor and associated interventions. *SMS may be scheduled or triggered by a delayed or missed dose; they may also involve 1- or 2-way communication.

We studied use of the evriMED electronic adherence monitor (Wisepill Technologies, South Africa), which transmits device opening data to a central server *via* cellular networks; data can be viewed as a digital display (e.g., adherence graphs) on a web browser or smart phone app (Figure 1). Multiple interventions can be associated with the monitors, such as data to inform counseling sessions in clinic. Short message service (SMS) reminders can also be sent to clients that may be one-way (i.e., an outgoing message) and/or two-way (i.e., invite a response with any questions). One- or two-way SMS notifications can also be sent to individuals identified as supporting the clients (e.g., family or friends; henceforth called social supporters). These SMS may be sent at a scheduled time or in response to a delayed or missed dose (e.g., 30 min after the anticipated dosing time). SMS were routed through a local technology solutions provider (Yo! Uganda Limited). Additionally, the device has an optional alarm that may be triggered at or before the anticipated dosing time.

### Study Design and Setting

In the pilot study presented here, we first implemented the evriMED monitor and associated interventions at the Kabwohe Clinical Research Centre (KCRC) in rural, southwestern Uganda. This clinic provides comprehensive primary healthcare services to >5,000 patients, including HIV care. It also has experience participating in research studies. We then assessed use of the monitors and associated interventions at the Kabwohe Health Centre IV Clinic (KHC-IV). This clinic is a more prototypic clinic for the region, focused primarily on routine care delivery for >2,000 patients.

### Participants

ART clients were eligible for participation if they were 18 years or older, received HIV care at one of the two above-noted facilities, and owned a cell phone; those who had participated in the earlier formative research were eligible. Enrollment was stratified with a goal of equal representation of early (<6 months) vs. established (>6 months) treatment experience and rural vs. peri-urban residence. Potential participants were approached as a convenience sample within these strata. Enrolled ART clients were then asked to invite a social supporter who knew their HIV status and owned a cell phone.

Healthcare administrators and clinicians who had participated in the formative research (15) were eligible for the current study and represented the following cadres: MoH officials, regional referral hospital administrators, district health officers, healthcare administrators, physicians, nurses, and ART adherence counselors. If the original participants were not available, others in the same role were invited to participate. The only exclusion criterion for any type of participant was the inability to provide informed consent.

### Study Procedures

#### Clinic Training

Consistent with the implementation goals of this study, Ugandan-based study staff (authors JT and ETu) led a training on use of the technology for each clinic in the manner of typical in-service trainings conducted by the Ugandan MoH. Research staff were then available to assist the clinical staff with operational issues (RB and JTu) and technical advice (JT and ETu).

#### Participant Data Collection

RB and JTu administered baseline questionnaires to enrolled ART clients and social supporters, using the local language (Runyankole) or English, as preferred. The questionnaires assessed the demographics and HIV-related clinical history of the ART clients and social supporters, as well as the ART client's structural barriers to clinic access (16), food insecurity (17), depression (18), alcohol use (19), beliefs about HIV, satisfaction with clinical care (20), and perceived HIV stigma (21). RB and JTu also administered an exit survey to ART clients to assess acceptability of the monitor and associated interventions. Questions focused on perceived ease of use, usefulness, problems experienced, and privacy and confidentiality concerns.

#### Monitor and Intervention Implementation

Clinic staff independently trained the ART clients and social supporters on the use of the monitor and asked them to select any (or none) of the available interventions. Participants were observed for a three-month period that included at least one routinely scheduled clinic visit according to Ugandan ART guidelines (22). Research staff only engaged with ART clients or their social supporters in the event of a technical problem with the monitors or associated interventions.

#### Implementation Metric Collection

RB, JTu, and JT collected implementation data, including the number of (1) devices used, (2) counseling sessions that occurred, and (3) functionality/technical problems experienced as feasibility metrics. They additionally conducted time and motion studies to assess the inner setting, noting which staff engaged with the technology and the time involved, comparing participants using vs. not using the monitoring and associated interventions.

#### Qualitative Interviews

After completion of the above-noted procedures at the first clinic (KCRC), JH, LG, and SA prepared a summary of the participant and clinic experiences that RB and JTu presented to a subset of the ART clients (flip chart format) and healthcare administrators and clinicians (text format; see Appendix 1a,b). ART clients were purposively selected to reflect roughly equal balance by gender and duration of experience with ART. Interviews were conducted in English or Runyankole in a private, quiet space. RB and JTu are both bilingual in English and Runyankole and trained in qualitative methods. They asked participants about views on the technology, perceived utility for routine care, concerns about implementation, recommendations for improvement, and perceptions of the intervention relative to other clinical programs or interventions (see Appendix 2). Interviews were conducted until thematic saturation was achieved.

### Analysis

We summarized quantitative data descriptively. Adherence reflects doses taken over the time monitored and was censored at death or study exit. Costs reflect salaries according to the Ugandan MoH and time recorded for effort spent on training and implementation of the monitors, associated counseling, and SMS. Costs solely related to study-related activities are not presented.

We analyzed qualitative data using an inductive, content analysis approach (23). LG, MS, and JH read the first 20% of transcripts to identify relevant content. LG and MS then formulated codes based on this content and assembled a codebook that was piloted and ultimately applied to the complete dataset. LG and MS performed the coding independently and disagreements were resolved through discussion. They entered data into Dedoose (version 4.12) to support the category development process, which consisted of characterizing core concepts, developing labels to represent the concepts, writing operational definitions, and selecting illustrative quotes as evidence from the interviews.

### Ethics

All participants provided written informed consent. This study was approved by the institutional review boards at the Mbarara University of Science and Technology, Ugandan National Council for Science and Technology, and Mass General Brigham. The study was registered with ClinicalTrials.gov (NCT03825952).

## Results

### Initial Training

The training at KCRC consisted of all 21 clinical staff who represented all cadres (i.e., nurses, counselors, doctors, pharmacists, and administrators). It was interactive and lasted approximately 3 h. The primary challenge involved accessing the internet on some of the clinical staff's phones, many of which were several years old and had damaged screens and/or keypads. During the session, it became apparent that focused training was needed for the two triage nurses identified to be responsible for use of the devices in routine clinical care. At KHC-IV, training involved an overview for all 11 clinical staff who represented all the above-noted cadres and lasted approximately 1 h. Two triage nurses underwent more intensive training for 3 h. Three of the four total triage nurses reported no difficulty with learning the procedures for registering ART clients and/or setting up the SMS interventions. One triage nurse required repeat training on proper phone number formatting and other data entry at registration.

### Participant Characteristics

A total of 51 ART clients were enrolled, 25 at KCRC and 26 at KHC-IV. All individuals approached for enrollment agreed to participate. Retention was high with 94% (48/51) completing the three-month follow-up period. One ART client at the KCRC site died from HIV complications shortly after enrollment; in KHC-IV, one participant moved away shortly after enrollment and was replaced, and one ART client at KHC-IV was lost to follow-up midway through the study.

As presented in Table 1, most clients (29/51; 57%) were male with a mean age of 34 years. Sixty percent of participants had a primary education or less, but 98% (50/51) were literate in Runyankole. Over half (30/51; 59%) were married and most were employed (46/51; 90%). Duration of ART use was equally split (25 were <6 months; 26 were >6 months). Roughly one-third (16/51) were taking efavirenz-based ART; most others took dolutegravir-based ART. Most clients had good immune function (average recent CD4 count 381 cells/mm^3^). Most (88%; 45/51) had disclosed their HIV status, and stigma in the form of negative perceived attitudes and disclosure concerns was moderate. Few structural barriers to care were reported. Food insecurity and depression affected one-fifth (10/51) and one-third (16/51) of clients, respectively. Nearly half (45%; 23/51) had problematic alcohol use. Clinic satisfaction was generally high, although more so in KCRC.

**Table 1:** ART client and social supporter characteristics.

**Characteristic**	**Kabwohe Clinical Research Centre**		**Kabwohe Health Centre IV**	
	**ART clients** **(*n* = 25)**	**Social** **supporters** **(*n* = 25)**	**ART clients** **(*n* = 26)**	**Social** **supporters** (*n* = 23)*
Female (vs. male)	10 (40)	13 (52)	12 (46)	16 (70)
Mean age (years)	38 (11)	42 (11)	31 (7)	35 (9)
Rural (vs. peri-urban/urban) residence	10 (40)	n/a	13 (50)	n/a
Highest education level^a^ None Primary (P1-P6) Secondary (P7-S6) Higher (>S6)	2 (8) 13 (52) 9 (36) 1 (4)	n/a	0 (0) 6 (23) 18 (69) 2 (8)	n/a
Literate in Runyankole (vs. not)	25 (100)	n/a	25 (96)	n/a
Marital status Married Widowed/divorced/separated Single	12 (48) 7 (28) 6 (24)	n/a	18 (69) 2 (8) 6 (23)	n/a
Relationship to ART client Spouse/partner Other family Friend Other	n/a	7 (28) 9 (36) 8 (32) 1 (4)	n/a	6 (26) 3 (13) 13 (57) 1 (4)
Employed (vs. not)	25 (100)	n/a	21 (81)	n/a
Living with HIV (vs. not)	25 (100)	13 (52)	26 (100)	23 (100)
Lowest CD4 count (cells/ml)	396 (255, 432)	n/a	348 (226, 452)	n/a
Most recent CD4 count (cells/mL)	414 (255, 599)	n/a	348 (226, 472)	n/a
Duration of ART use <6 months (vs. >6 months)	12 (48)	n/a	13 (50)	n/a
ART regimen backbone Efavirenz Dolutegravir Other	12 (48) 10 (40) 3 (12)	n/a	4 (15) 21 (85) 0 (0)	n/a
Disclosed HIV status (vs not disclosed)	22 (88)	n/a	23 (88)	n/a
HIV stigma^b^ Negative attitudes	
High Moderate Low	11 (44) 8 (32) 6 (24)	n/a	4 (15) 8 (31) 14 (54)	n/a
Disclosure concerns High Moderate Low	7 (28) 7 (28) 11 (44)	n/a	9 (35) 10 (38) 7 (27)	n/a
Structural barriers scale^c^	2 (0, 8)	n/a	4 (0, 6)	n/a
Food insecure (vs not)	7 (28)	n/a	3 (12)	n/a
Probable depression (vs not depressed)	9 (36)	n/a	7 (27)	n/a
Alcohol use problematic (vs not problematic)	11 (44)	n/a	11 (42)	n/a
Clinic satisfaction^d^	3.8 (3.5, 3.9)	n/a	3.0 (3.0, 3.3)	n/a

*Data reflect N (%) or median (interquartile range [IQR])*.

^*^Four clients used the same social supporter. ^a^P1-P6 are typically for students 5–11 years old; P7-S6 are typically for students 12–18 years old. ^b^The stigma scale is recorded as 1–4 with higher score indicating more stigma. ^c^The structural barriers scale reflects an added value of responses with values of 0 to 4 over 13 questions with a higher score indicating more barriers. ^d^The clinic satisfaction scale reflects a median value of 1–4 over 13 questions with a higher score indicating more satisfaction. “n/a” = not applicable or not assessed.

All ART clients enrolled in the study with a social supporter. Notably, four participants at KHC-IV selected the same clinic volunteer as their social supporter. Sixty percent (29/48) of social supporters were women with a mean age of 39 years. Roughly one-quarter each were spouses/partners (13/48) and other family (12/48). Three-quarters (36/48) of the social supporters were also living with HIV, including all in KHC-IV. All social supporters living with HIV were taking ART.

### Monitors and Associated Intervention Functionality

We attempted to import 35 devices; however, only 25 were obtained due to loss during the shipping process. Eight technical errors occurred. Three devices stopped working for unclear reasons, of which two resumed function after restarting and one had to be replaced. Two SIM cards had to be re-positioned, two devices were stolen, and one participant's alarm was activated in error. The three devices that were non-functional or stolen were replaced. Most batteries lasted the full 3-month study period, although two required additional charging. Choice of interventions associated with the adherence monitors is shown in Table 2. All clients chose to receive SMS reminder (daily and/or triggered), and most also chose to send an SMS notification to the social supporter and an alarm. All clients were able to contact the triage nurses or counselors as desired, although the frequency of these calls was not tracked.

**Table 2:** Intervention selection, functionality, and adherence outcomes.

	**KCRC** **(*N* = 25)**	**KHC-IV** **(*N* = 26)**
Interventions*		
•SMS reminders to ART clients		
∙ Triggered only	17 (68%)	4 (15%)
∙ Scheduled daily only	5 (20%)	9 (35%)
∙ Both	3 (12%)	13 (50%)
•Triggered notifications to social supporters	21 (84%)	19 (73%)
•Alarms (daily)	22 (88%)	22 (85%)
Functionality		
•Daily SMS (2,892 days of follow-up for 29 ART clients)		
∙ Sent as expected	2,715 (94%)	
∙ Not sent due to system errors	177 (6%)	
∙ Triggered SMS (3,575 days of follow-up in which 1,674 SMS should have been sent to 35 ART clients and 33 social supporters)		
∙ Sent as expected	1,086 (65%)	
∙ Sent unnecessarily	588 (35%)	
∙ Due to poor cellular network**	532 (90%)	
∙ Due to system errors	56 (10%)	
Adherence over 3-month follow-up period		
Mean (standard deviation [SD])	93% (SD 16)	94% (SD 27)
Number clients with 7+day interruptions	3	1

^*^Participants could choose multiple interventions; ^**^Poor cellular network resulted in the server receiving data from an on-time opening >30 min late.

SMS functionality is also shown in Table 2. Most SMS were sent as expected, although many triggered SMS were sent unnecessarily, primarily due to poor cellular network (i.e., the device was opened on time but the server received that data >30 min late). The most common technical problem arose from the one triage nurse who had challenges with training; she made errors in entering the desired SMS schedule at enrollment, resulting in both missed days of monitoring and overlapping dosing schedules. Error rates were similar by site and type of recipient. Most participants (45/51; 88%) experienced some SMS errors. Two social supporters received daily SMS as an error for 33 days. An additional challenge occurred with “anti-SPAM” legislation that initially blocked all SMS. Clients, however, were able to voluntarily unblock the SMS.

### Clinic Experience

In both clinics, the triage nurses and ART adherence counselors used their personal smart phones to access the adherence data (reports could not be printed). Additional targeted training was provided as needed over the course of participant follow-up for approximately 5.5 h total. Forty-three time and motion studies were conducted among study participants and other ART clients (17 in KCRC and 26 in KHC-IV) and indicated that triage nurses played a key role in using the devices in clinic; their review of the data determined who needed in depth counseling and who could skip it entirely. ART adherence counselors also used the data to inform counseling sessions; nurses and doctors did not. An additional 4 min on average were spent per client using the adherence monitoring intervention compared to routine care (7 and 1 min in each site, respectively).

### ART Client Experience and Acceptability

Clients were seen according to their routine clinic schedule, which varied depending on their prior duration of treatment. The median number of visits was two per client, ranging from one to three. At study exit, most (90%; 43/48) clients reported that their counselors reviewed the monitor data with them during one or more routine visits; 32/48 (67%) reviewed the data at all visits. The most common reason for not reviewing the data was poor internet connectivity, followed by time. As shown in Table 2, overall average adherence during the 3 months of follow-up was 94%; however, four clients had gaps in adherence of >7 days (three in KCRC and one in KHC-IV).

As indicated in Table 3, acceptability was high with all but one client reporting that the monitor and data used for counseling were very useful; 92% (44/48) also found the SMS very useful. Nearly all participants reported no problems with the monitor or the SMS. Thirteen percent (6/48) of clients were worried about others seeing the monitor, while 4% (2/48) were worried about someone seeing the SMS. One participant reported a problem with storing the monitor.

**Table 3:** Acceptability of the monitors and associated interventions.

	**KCRC** **(*N* = 24)**	**KHC-IV** **(*N* = 24)**
How useful was the monitor?		
Not at all Somewhat useful Very useful	0 (0) 0 (0) 24 (100)	1 (4) 0 (0) 23 (96)
How useful were the counseling sessions using the monitor data for taking ART?		
Not at all Somewhat useful Very useful	0 (0) 0 (0) 24 (100)	0 (0) 1 (4) 23 (96)
How useful were the SMS from the monitor for taking ART?		
Not at all Somewhat useful Very useful Not applicable*	0 (0) 0 (0) 20 (83) 4 (17)	0 (0) 0 (0) 24 (100) 0 (0)
Did you have any problems in using the monitor?		
Not at all Some problems A lot of problems	24 (100) 0 (0) 0 (0)	22 (92) 2 (8) 0 (0)
Did you have any problems receiving the SMS from the monitor?		
Not at all Some problems A lot of problems	23 (96) 0 (0) 1 (4)	24 (100) 0 (0) 0 (0)
Were you worried that anyone would see the monitor?		
Not at all Somewhat worried Very worried	21 (88) 3 (13) 0 (0)	21 (88) 1 (4) 2 (8)
Were you worried that anyone would see the SMS from the monitor?		
Not at all Somewhat worried Very worried	23 (96) 1 (4) 0 (0)	23 (96) 0 (0) 1 (4)
Did you have trouble finding a place to store the monitor?		
Yes No	0 (0) 24 (100)	1 (4) 23 (96)

*Data are missing for 1 participant who died (KCRC) and 1 who was lost to follow-up (KHC-IV)*.

^*^Participants with no missed doses who had selected triggered SMS.

### Cost

One-time costs included the 28 monitors used in the study, ($25 each) plus license fees, SIM cards, battery chargers, and shipping, totaling $2,170. On-going costs included SMS and data hosting fees of $1,020 per year. An additional $438 was spent on the initial training, accounting for staff time and refreshments, plus $3,190 for technical support (160 h at approximately $20 per hour) over the course of the study. If costs were extended for an anticipated 3-year lifetime for the device and assuming technical support needs would decrease by 75% in subsequent years, they would average a total of $139 per client per year.

### Perceptions of Implementation

After reviewing the summary of participant and clinic experiences from KCRC (Appendix 1a,b), we interviewed 10 ART clients, of whom six were men and four women; six had taken ART for >6 months, while four had taken ART for <6 months. We also interviewed 19 healthcare administrators and clinicians, consisting of the following cadres: four MoH officials, two district health officers, two healthcare administrators, three physicians, two clinic officers, three nurses, and three adherence counselors. Reflections are presented here by CFIR domain (see Table 4).

**Table 4:** Participant quotations from qualitative interviews according to the CFIR.

**Domain**	**Participant quotations**
**Intervention**	
Benefits from the technology	•“When we were taking [ARVs] without these monitors, we would forget to take our drugs or even forget the days for our refills. But when we got these devices, you can easily realize that your drugs are finished and that you should go for your drug refill at the clinic. This is the value that the monitor has which other programs don't have.” (34-year-old, male ART client) • “In ordinary settings, clients have learned how to do pill count, so they know how to hide the reality from the counselor or from the clinician. If you depend purely on pill counting, then this client will not be given the support they deserve… but the device indirectly monitors them and prepares counselors and clinicians to offer these people additional support that people need.” (43-year-old, male MoH official)
Suggested modifications and concerns	•“There is only one challenge: there are many people in our population who actually cannot read SMS, so the SMS platform may work but will not serve 100% of the population.” (43-year-old, male physician) • “If my phone does not have network, you yourself who is monitoring me, you are not going to get whether I have taken the drug or not. And even if it is not there, the reminder will not come.” (42-year-old, female District Health Officer) • “If this monitor could be modified to put the number of pills you have removed and swallowed and the number of the pills remaining in the device, it could even be better than it is now and help more.” (60-year-old, female ART client)
**Clinic setting**	
Optimized clinic flow	•“For these triage nurses, they can see if you have been adhering well, if you have no problem, you just go and get your refill drugs and go without undergoing adherence counseling. It saves time.” (27-year-old, female nurse) • “According to the results I have seen, the monitoring device is helping in ART adherence… clients are helped and reminded every time to take their ARVs on time, and then the clinical team, is also helped to get the information if the patient is taking the drugs as expected because as we have seen, at every visit, they review the information/data that is in the device which shows them how this patient has been taking the ART treatment.” (33-year-old, female nurse) • “It will ease the system of providing care, it will help us to monitor adherence and provide quality care. So I think it is very valuable compared to other programs.” (26-year-old, female adherence counselor)
Logistical benefits	•“If you have data from our monitors, then you can probe into the patient, and you can be able to fill in your documents, and you improve documentation in your routine practice.” (30-year-old, male physician) • “It can only reduce on the costs of the clinic. We have been sending messages, and an SMS message from a clinic phone is also at cost… if the client fails to come, then it means you have to arrange home visit, so all these costs are avoided for as long as this device is used correctly… Now at program level and policy, it saves the country resources, because if you have prevented resistance then it means we can deploy those regimens for quite long time.” (45-year-old, male MoH official)
Additional clinic needs	•“Reading the monitors, you're using people's smart phones. Do public servants have smart phones? Of course not all of them. Reading the monitors requires internet. Do hospitals or health centers in Uganda have provision of internet at all facilities? Using the monitors, I have seen you have additional support and additional counseling. (43-year-old, male MoH official) • “Majorly I think it [additional support] is just providing the monitoring device and giving the training because even if you're 2 clinicians or 2 staff, once you are trained, you can use it because the resources are never enough, and those that are available, you use them the way they are.” (35-year-old, female adherence counselor)
**Individual**	
Improved adherence	•“It helped me to love my life more and increased my commitment to taking my drugs to remain healthy… I liked the monitors for teaching us time-keeping and taking our drugs at the time we are supposed to take them.” (42-year-old, male ART client) • “I like the fact that majority of the clients consented to get a reminder, meaning that actually they perceived that they are likely to forget. So it is addressing something very key.” (55-year-old, male district health officer) • “[It] gave joy and morale to my social supporter because he could not worry about reminding me since my adherence was good and I was taking my drugs well because of the reminders which came with the device, especially the alarm. My social supporter knew that the alarm would always remind me and that made him always feel settled.” (33-year-old, male ART client)
Trust and morale	•“So if a client knows that I am given this and know that it is quality, the client knows that what they are doing for me is really good and it gives them morale as individuals to know that I am cared for and I am moving somewhere. It gives hope.” (26-year-old, female adherence counselor) • “It led to good and improved interactions with the counselor because when you find that your counselor knows that you don't take your drugs well or on time, and he/she takes the responsibility and advises you to always take your drugs well and on time, and the advice given is always good/well intentioned. Because we get the advice and try to follow that advice, and when you find that someone else cares and loves your life and health, it's a good thing.” (29-year-old, female ART client)
Social determinants of health	•“SMS has no problem. Because even these telecommunication companies like MTN and Airtel send messages always. When I receive a message and even my neighbor can't know what the message is about. I read my message from my phone without anyone even that next to me knowing what the message is about.” (42-year-old, male ART client) • “Provide them to all patients that are on chronic care in a set up. Then you avoid the level of “that device is for HIV.” So if you come at the clinic and you have HIV, we give the device, and when you come [for] diabetes we give you the device so that we dis-stigmatize.” (43-year-old, male clinician)
**Outer setting**	
Cost-benefit ratio	•“You see to access real time data from the monitors, you have to use internet, and internet in this part of the world again is not something very affordable, and it is a strain that we have to meet every day for us to run the clinic, and so the fact that this also have to use internet it may be a little bit increasing expenses of the clinic.” (29-year-old, male clinician)
	•“But we would want you in your analysis to prove to us that actually sending an SMS creates a significant change in what is expected. So provide us with more information that is cost-benefit.” (43-year-old, male MoH official) • “Once we see the cost of deploying this are much less than the cost of not deploying them due to non-adherence, then we will be able to say precisely that there is need for us to prioritize… And someone needs to try it for the health economist and we need it analyzed and displayed to us, the policy makers, and that is the argument that is going to made by the minister when we are going to present this device… so the government cannot fail to mobilize recourses for the rollout of this as long as there is buy in and consensus at national level.” (45-year-old, male MoH official)
Evidence needed to implement	•“I noticed that clients are very clever; those who don't want to adhere can open the monitor, pick medicine, and throw it away and don't take it. I would want, as part of your analysis, to include some biological sample to see the effectiveness of the technology—how it relates to the findings like on blood levels.” (43-year-old, male MoH official) • “Yeah, I am happy with the provisional results. I have read through and I realized that the study was very successful and especially to us, the clinical team, it was very helpful in a way that adherence of clients was being monitored on real time and the information we could get would help to inform the information given during adherence and clinical assessment and that one would improve adherence outcome of clients. So I am happy with the results and I wish it could be rolled out for clinical work.” (39-year-old, female clinic administrator) • “I can [influence the decision to make this technology available to patients] if more information is brought on board from the pilot focusing on categories of patients…. or qualitative strong feedback…from the users and advocacy from the civil society, the PLHIV community, advocating for it and pushing for monitors as a tool which is helpful.” (55-year-old, male district health officer)
**Implementation process**	
Implementation	•“I think it depends on those top people. If they accept and they agree to fund the intervention, I think it can work. But if you say that we the Health Center IV or the In-charge or the health workers improvise and put the money, it cannot work. But if the MoH can support, then it can work and they can afford it.” (27-year-old, female nurse) • “Researchers come and do research, they get information, and what they do is publish it in papers and the Member of Parliament never knows about that. So we will need that one–the results that we get from the research side– we share them with clinical caregivers and clinical program manager but also with country program and policy makers, so we need to have platform and benefits are known by the researchers who should share it with clinical care givers and government leaders so that we can get a buy in.” (43-year-old, male clinician)
On-going support	•“We need some follow up, mentorship, and coaching because there are so many hurdles that may come in place. Because these are new machines and they may develop any problem.” (44-year-old, male MoH) • “Basically, what is needed is to train the available staff and make them know. They will automatically do the work because they have been there. They will just switch to the new system.” (26-year-old, female adherence counselor)
Target population	•“It wouldn't be given to everybody; it will be given to those who are struggling with adherence. It should be used in the context of viral load monitoring.” (55-year-old, male district health official) • “We can also test it in different socially communities like fishermen, taxi drivers, and see if we can get similar results so from you, you can be able to tell whether it can work across social profiles.” (43-year-old, male clinician) • “I would want [the devices] to be used for life or the whole of their treatment lives on ART… because they help the clients to take their drugs well by reminding them when it's time to take drugs.” (47-year-old, female ART client) • “It should be given to everyone at the time of initiating ART. It should be given to every client who is initiating ART so that it can help to take away their worries and fear and put them on line of good adherence right away from the start.” (33-year-old, male ART client) • “Everybody [should use monitors], especially those with chronic disease who need to be reminded to take their medicine especially at specific times. I think it's a good reminder tool, whether it's taking your tablet for BTS or for hypertension—anything.” (43-year-old, male clinician)

#### Intervention

Participants generally liked the monitors and SMS and felt they improved adherence through desired accountability. Although opinions differed on the accuracy of the data and potential for using the devices as expected, participants generally agreed that the objective information added value for clinical care.

Some participants had concerns about the technical function of the monitors and SMS, including faulty batteries. They were worried that poor cellular network would prevent reminders from coming as expected. Participants suggested modifications, including voice recordings and/or images for those with low literacy and modifying the monitor to track the number of pills taken out.

#### Clinic Setting

Participants reported that the adherence data qualitatively enhanced the conversations between adherence counselors and clients. For example, data demonstrating patterns of adherence behaviors allowed for more focused counseling conversations. Clinic staff also noted that knowing the data ahead of time allowed them to provide better care through longer counseling when needed.

Healthcare administrators and clinicians felt the intervention had other logistical benefits as well. The automated reminder messages reduced the time they spent calling or visiting ART clients, lightening their burden. They stated that the monitors additionally improved documentation and record-keeping, thus providing logistical benefits to the clinic and reduced costs.

Participants did not express concerns about the monitor data delaying them in clinic; however, opinions differed on the need for additional staffing and resources, including phones for professional use, technical support, and counselors.

#### Individual

Nearly all participants emphasized that the monitors and system of reminders built better adherence habits, while simultaneously reducing worries about defaulting on treatment. They also felt the intervention reduced the burden on both ART clients and their social supporters. Many saw the data as building trust within the client-counselor relationship, placing them on the same team and improving counselor morale. ART clients liked the technology, because it enabled them to demonstrate high adherence and thereby please their clinicians.

Despite anticipated concerns about the influence of social determinants of health in the formative interviews, relatively few participants mentioned poverty, stigma, and privacy. Most felt these issues could be managed through careful program implementation and individualized strategies.

#### Outer Setting

The most prevalent concerns from the outer setting were related to the cost implications of the technology, which could be positive or negative. Healthcare administrators and clinicians wanted to know the cost-benefit ratio, including impact on resources and staffing. These factors would guide public policy decisions regarding uptake of the monitors and associated interventions.

In deciding whether or not to implement this technology, some participants wanted a large, rigorous, study in multiple parts of the country with clinical outcomes, objective measures, and a comparison to standard counseling. Others wanted more qualitative evidence from communities of people living with HIV, and still others felt this pilot was adequate.

#### Process

Nearly all participants stated that the MoH would need to support and fund the implementation process. One healthcare administrator emphasized the need for a platform to translate research findings into practice. Healthcare administrators and clinicians stressed the importance of initial and ongoing in-service training to support proper use of the intervention, although some felt staff would adopt it readily.

Beliefs on the appropriate target populations for the intervention varied widely, ranging from people living with HIV who failed first-line ART to members of key populations to anyone with chronic disease. Some ART clients also recommended using it for short time periods to establish the habit of adherence, while other ART clients and clinicians recommended people might benefit from them over their whole lives.

## Discussion

This pilot mixed methods study presents the pragmatic experience of implementing relatively low cost, real-time electronic adherence monitors and associated interventions for routine ART delivery in rural southwestern Uganda. Implementation was accomplished by clinic staff with minimal support from the study team. Feasibility was generally high with few, mostly temporary problems with the adherence monitor functionality. SMS reminders to ART clients and social supporters were transmitted reliably when sent daily; however, limitations in cellular network and some system errors resulted in SMS being triggered unnecessarily. Acceptability for both the monitors and SMS was very high with a minority expressing concerns about privacy. Perceptions of healthcare administrators, clinicians, and ART clients about implementation of the technology were generally favorable. They felt it had a positive influence on adherence, optimal clinic flow, and relationships in the clinical setting. Participants emphasized the importance of demonstrated benefit relative to cost and the need for buy-in from the MoH for actual implementation. Opinions differed about on-going support needs and target populations.

Although this study was small, the setting, participants, and types of ART were largely typical for HIV care in rural sub-Saharan Africa; our findings thus provide important insights into the use of this type of intervention in routine care. Moreover, experiences were generally similar in the two clinics regardless of prior experience with research. Well-known adherence challenges, including food insecurity, depression, alcohol use, and stigma, were present in the study population, indicating their potential need for adherence monitoring and support. Importantly, the relatively low levels of education did not seem to present a barrier to acceptability and use of the technology, and social determinants of health (e.g., poverty) were not prominent concerns in our qualitative interviews. That said, reported barriers to care were relatively low, and experiences for people with HIV who are less engaged in care may differ. Notably, the majority of participants in our study were men, while women comprise the majority of people living with HIV in sub-Saharan Africa. Additional studies in diverse settings and populations will therefore be important for understanding the potential of this technology broadly.

Both clinics chose to implement the technology among a small number of clinical staff (i.e., triage nurses and ART adherence counselors) who have more time and availability relative to physicians and nurses. Given the healthcare worker shortages across sub-Saharan Africa (24), the central role played by the triage nurses and adherence counselors bodes well for scalability of this technology. That said, the challenges in training one triage nurse and the subsequent impact on SMS errors suggest the need for careful skill assessment and/or a simpler user interface with the technology. Additionally, the impact on length of visits was minimal, and participants commented favorably on the potential of data-informed counseling to improve the time spent in clinic, including type of counseling and relationships between ART clients and clinic staff. These features are consistent with differentiated care models, which have been shown to improve clinical care (25). Notably, none of the participants described the monitors or associated interventions as disruptive or a distraction, as has been seen with other forms of technology used for adherence monitoring, such as cell phone-based self-report for the treatment of tuberculosis (26). This combination of simple, “smart” pill boxes for data-informed counseling and options of SMS and/or alarm support appears to have potential for improved overall patient-oriented clinical experiences.

Our study focused on assessment of implementing real-time adherence monitoring and associated interventions, rather than the effects on ART adherence itself which have been published in the literature (7–12). Nonetheless, we did identify potentially clinically meaningful episodes (i.e., >7 days) of non-adherence among four of the 51 study participants (8%) over just 3 months of follow-up per participant. Although these participants had generally high overall adherence, the risk for loss of viral suppression has been shown to increase with each consecutive day of non-adherence (27). The ability to detect these sustained gaps, trigger interventions, and tailor adherence counseling suggests potential for impact on long-term adherence, sustained viral suppression, and thereby use of low-cost, first-line ART.

Cost is a major consideration when assessing interventions for routine clinical care, particularly in light of limited resources. Indeed, participants indicated the cost-benefit ratio would be a main driver of the decision to take up this technology. The costing in this study was limited and could not evaluate cost-effectiveness; however, we tracked the primary costs incurred with clinical use of the technology. Although the monitors were only $25 USD each, additional one-time and on-going fees resulted in an estimated cost of $139 per client per year. An analysis of potential ART adherence monitoring interventions for sub-Saharan Africa found that up to $50 per person-year could be cost-effective, primarily based on differentiation of care and varying based on the availability of viral load testing (28). Although our estimates exceed this threshold, many costs would reduce considerably with economies of scale and cost-effectiveness should be explored in future studies involving high volume clinics.

This study has important limitations. Principally, as noted above, the study was small in scope and focused on short-term feasibility, acceptability, and perceptions of implementation. That said, the engagement of two clinics reflective of ART care in rural, sub-Saharan Africa with minimal support from research staff is a major strength. Additionally, our use of the CFIR provides broad opinions across the healthcare system, which are important for considering potential effects.

In sum, this study shows promise for the implementation of relatively low cost, real-time electronic adherence monitors and associated interventions for ART delivery in sub-Saharan Africa. Feasibility and acceptability were high with favorable impressions of impact on adherence and clinic experiences. Future work should involve longitudinal follow-up of diverse populations, clinical outcomes, and detailed cost-effectiveness analysis to help drive policy decisions around the uptake of this technology for routine clinical care.

## Data Availability Statement

The raw data supporting the conclusions of this article will be made available by the authors, without undue reservation.

## Ethics Statement

The studies involving human participants were reviewed and approved by the institutional review boards at the Mbarara University of Science and Technology, Ugandan National Council for Science and Technology, and Mass General Brigham. The patients/participants provided their written informed consent to participate in this study.

## Author Contributions

JH and SA conceived of the study. RB and JTu collected data for the study. JTi and ETu provided technical oversight. LG and ETi provided program management. LM supported study implementation. JH, LG, and MD conducted the qualitative analysis, while JH and NM conducted the quantitative analysis. JH wrote the first draft of the manuscript. All authors edited and approved the final manuscript.

## Funding

This study was funded by the US National Institute of Mental Health (K24MH114732). Neither the funder, nor the manufacturer of the technology studied (Wisepill Technologies), had any influence on the design, implementation, or data interpretation in this study.

## Conflict of Interest

The authors declare that the research was conducted in the absence of any commercial or financial relationships that could be construed as a potential conflict of interest.

## Publisher's Note

All claims expressed in this article are solely those of the authors and do not necessarily represent those of their affiliated organizations, or those of the publisher, the editors and the reviewers. Any product that may be evaluated in this article, or claim that may be made by its manufacturer, is not guaranteed or endorsed by the publisher.
