# Supplementary material for: Implementation of Electronic Adherence Monitors and Associated Interventions for Routine HIV Antiretroviral Therapy in Uganda: Promising Findings
Source: Front Digit Health. 2022 Jul 22;4:899643. doi: 10.3389/fdgth.2022.899643 (PMC9354256; doi:10.3389/fdgth.2022.899643)
Supplement: Supplementary file 1 [file Data_Sheet_1.docx]

**Appendix**

Appendix 1a. Summary of the participant and clinic experiences at KCRC: The flip chart used with ART clients


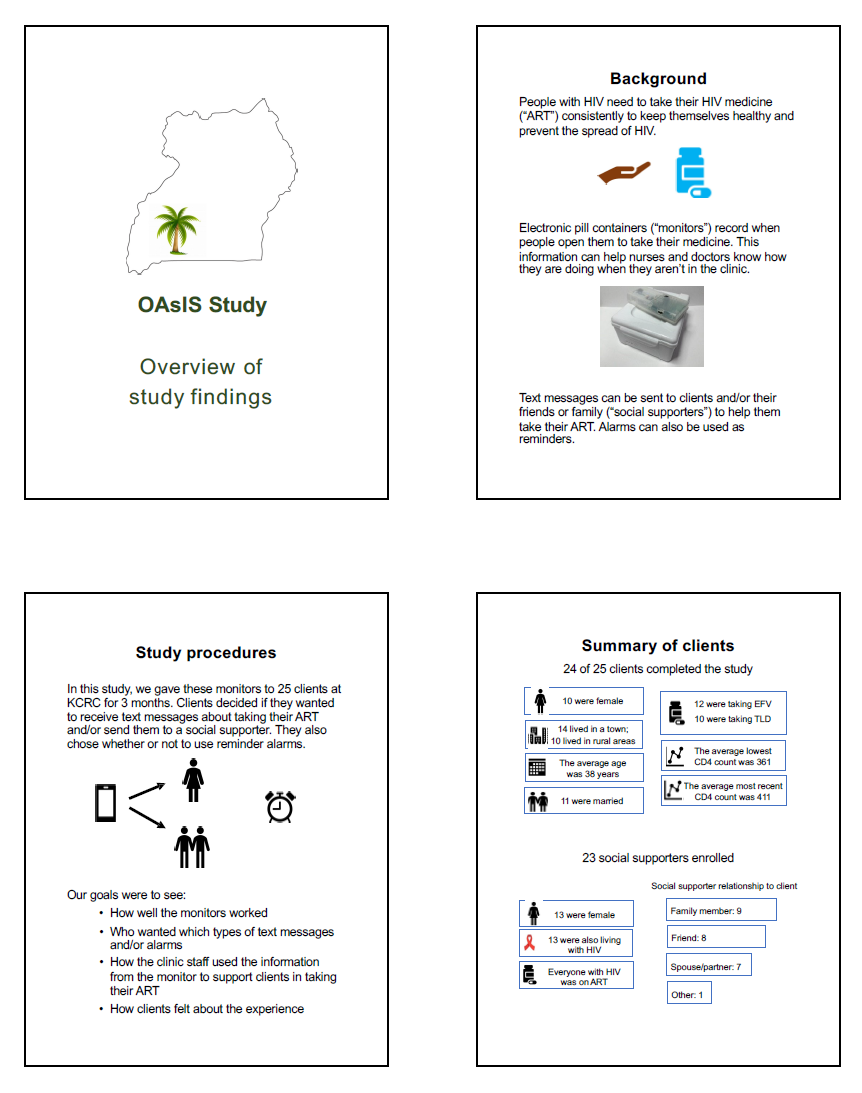


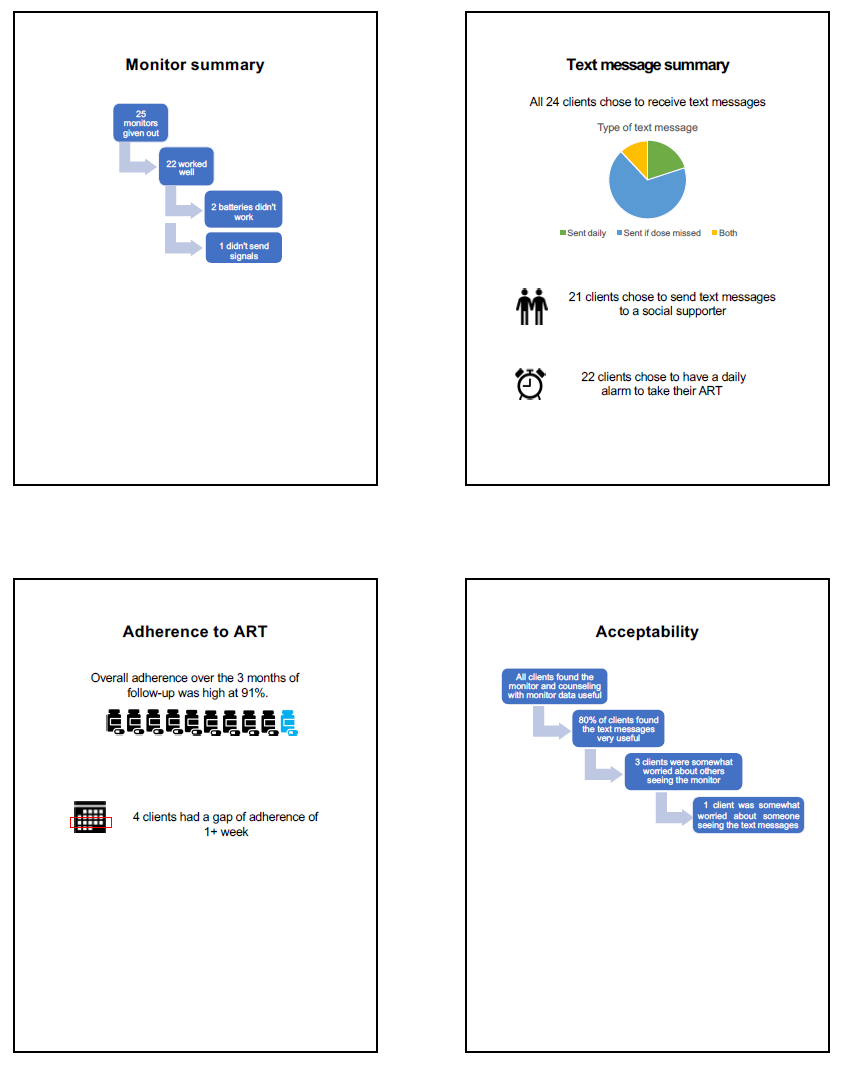


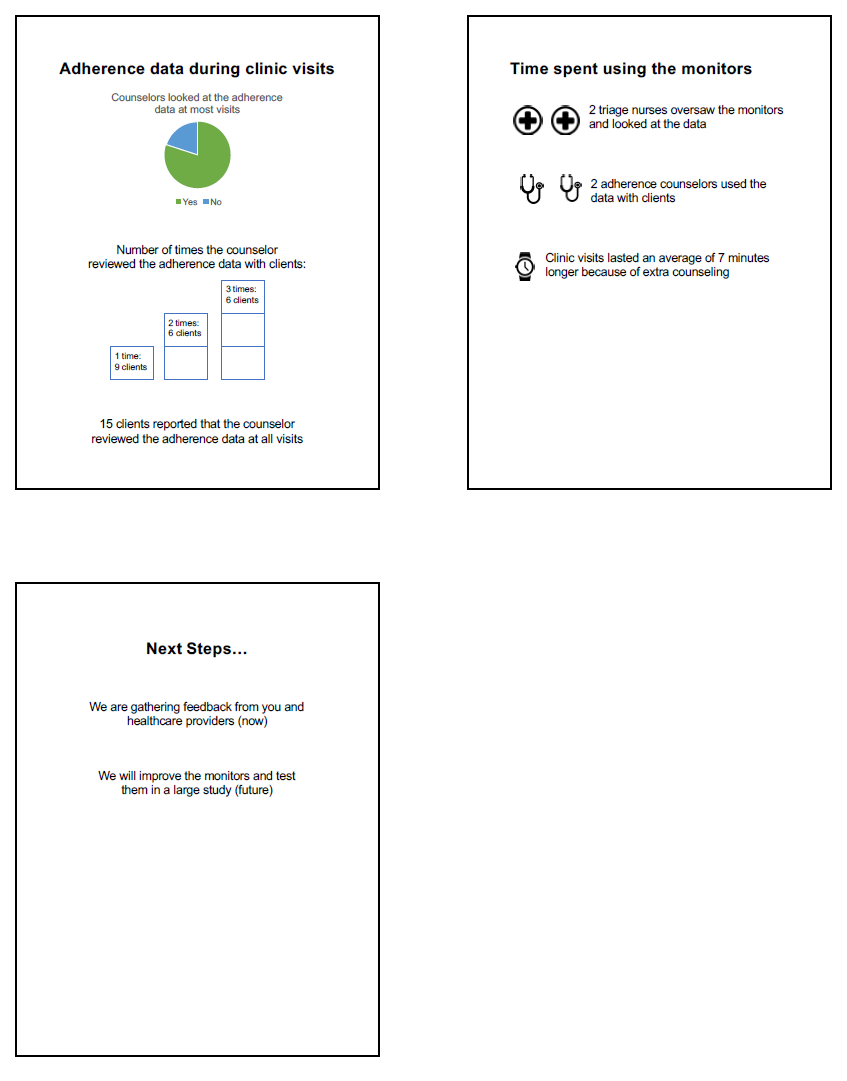


Appendix 1b. Summary of the participant and clinic experiences at KCRC: The written document used with healthcare administrators and clinicians

**Summary of initial experiences with the Medication Event Reminder Monitor**

**and associated interventions in routine care for people living with HIV**

**Study summary**

High, sustained pill taking (i.e., adherence) is critical for achieving the individual and public health benefits of HIV antiretroviral therapy (ART). Electronic pill boxes provide a detailed understanding of adherence and can enable real-time interventions. Research has shown the benefit of these devices (1-3); however, their use to date has largely been confined to research. A low-cost device called the Medication Event Reminder Monitor (see photo) has recently become available and may have applications for routine care. Estimated cost for 12 months of use is ~100,000 UGX ($27). It can be combined with other interventions, such as SMS reminders to ART clients and the people who support them, as well as counselling tailored to the individual’s pill taking history.


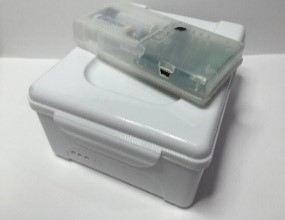
OAsIS is an implementation science-driven assessment of strategies to optimize uptake of the monitors and associated interventions for routine, clinical delivery of ART in Uganda. It is being conducted at the Kabwohe Clinical Research Centre (KCRC). The study consists of two aims. In Aim 1, we conducted formative interviews with a variety of healthcare administrators, clinicians, and ART clients to design a preliminary implementation strategy. In Aim 2, we are using an iterative approach to optimize that strategy. This report describes our initial experiences with implementation.

**Timeline overview**

We completed the formative interviews (Aim 1) in late 2018 and trained the KCRC staff to use the MONITOR and associated interventions in February 2019. ART clients were then followed for three months each between March and September 2019.

**Clinical staff training**

The training involved all clinical staff, consisting of 21 individuals and representing all cadres (e.g., nurses, doctors, pharmacists, administrators). The training lasted ~3 hours and was generally considered very informative. The primary challenge involved accessing the internet on all participant’s phones.

**Study participants**

A total of 25 ART clients were enrolled. Half of clients had recently initiated ART, while the other half had been taking ART for more than six months. Most clients (60%) were male with an average age of 38. Nearly half were married. Education was moderate and literacy in Runyankole was 100%. All clients were employed. Roughly half were taking efavirenz-based ART; most others took dolutegravir-based ART. Only 1 client took twice daily medications. Most clients had good immune function (average recent CD4 count 461 cells/mm^3^) and all but 3 had suppressed virus.

A few structural barriers to care were reported and clinic satisfaction was high. Food insecurity and depression each affected ~1/3 of clients. Nearly half (44%) had problematic alcohol use. Stigma in the form of negative perceived attitudes and disclosure concerns were moderate, but most (88%) had disclosed their HIV status to 1+ people. About 1/3 of clients had concerns about toxicity with ART, but nearly all found it proven to be effective, were sure it has a positive effect on them, and felt adherence was important even in the absence of symptoms.

All ART clients enrolled in the study with a social supporter who assists them with ART and HIV care. Half of the social supporters were women and the average age was 41. Roughly 1/3 each were spouses/partners, other family, or friends. Half of the social supporters were also living with HIV, all of whom were taking ART.

**Intervention experience**

We had initially planned to enroll 35 ART clients but were only able to import 25. Three of the devices (12%) had technical problems, consisting of faulty batteries. Functional batteries lasted the full 3-month study period.

Clients chose the following interventions to use along with the monitors: 5 (20%) daily SMS reminders, 17 (68%) SMS reminders triggered by a missed or delayed opening of the MONITOR, and 3 (12%) chose both. Additionally, 21 (84%) chose to send SMS notification to a social support triggered by a missed or delayed opening for the monitor, and 22 (88%) opted for a daily alarm. The SMS generally worked well with SMS being sent as planned for 99% of the daily messages and 96% of the triggered messages; only 81% were sent when both options were chosen. The major challenge with the SMS was “anti-SPAM” legislation, which initially blocked all SMS. Clients, however, were able to voluntarily unblock the SMS. Most had no problems thereafter, although a few were confused by true SPAM SMS coming to them from outside of the study.

**Client experience**

Of the 25 clients enrolled in the study, 24 (96%) completed the 3 months of planned follow-up; 1 client died shortly after enrollment due to illness. Clients were seen according to their routine clinic schedule, which varied depending on their prior duration of treatment. The median number of visits was 2 per client, ranging from 1-3. Most (88%) of clients reported that their counsellors reviewed the monitor data with them during 1+ routine visits; 15 (60%) reviewed the data at all visits. The most common reason for not reviewing the data was poor internet connectivity, followed by time.

Overall average adherence during the 3 months of follow-up was 91%; however, 3 clients had gaps in adherence of 7+ days. Acceptability was high with all clients reporting that the monitors and the data for counseling are very useful; 80% also found the SMS very useful. No problems with the monitors or the SMS were reported; 3 (12%) of clients were somewhat worried about others seeing the monitor, while 1 (4%) was somewhat worried about someone seeing the SMS. None were very worried about either technology. No clients reported a problem with storing the monitor.

**Clinic experience**

We conducted structured clinic observations (i.e., time and motion studies; Table 5) to assess how and where the monitor was used; 17 were done before and 21 (11 with study participants, 10 with clinic attendees) were done during the study. Based on this information, the clinic assigned responsibility for the monitor and its associated interventions to the 2 triage nurses who greet all ART clients. These nurses accessed the monitor data on their personal cell phones to determine if counselling was needed or not. If so, 2 adherence counsellors also accessed the monitor data on their personal cell phones and used it to guide the counselling session. Overall, routine clinic visits lasted slightly longer with the monitors with a median of 48 minutes at baseline and 55 minutes during the study. The difference was largely due to time spent with adherence counselors.

**Summary/Key points**

- **In a prototypical clinic, we successfully implemented the monitors and associated interventions among most clients who reported a high degree of acceptability.**
- **Training may work better as a brief informational session for all staff, reserving detailed training for those directly involved with the technology (triage nurses and adherence counselors).**
- **We encountered minor technical challenges that could be readily overcome.**
- **Adherence was generally high. However, the presence of >1-week adherence gaps in 4 (16%) clients indicates the potential value of the intervention.**
- **The data from the monitors were used to support counseling at most visits with minimal impact on overall clinic flow.**

**Future steps**

We are currently gathering feedback on our initial experiences from healthcare administrators, clinicians, and ART clients. We will then improve the way the monitors and associated interventions are used in the clinic and repeat our assessment. Costing analyses are also underway.

We would now like to get started with the interview. Do you have any questions before we begin?

Appendix 2. Qualitative interview guide– ART Clients

**READ:** I would now like to learn about your experience and reactions to the monitor and associated interventions used in this study.

- 1. Please tell me what you think of the monitoring device (now)? What do you like? What do you not like?

*Probe on recommendations for device improvement*

*If interviewing a participant who was interviewed in Aim 1, probe on significant likes/dislikes*

- 1. In what ways do you think the monitor was helpful for routine clinical care?

*Probe on individual benefits for patients, benefits for clinic flow and outcomes*

- 1. What are your concerns about the way the monitor was used for routine clinical care?

*Probe on individual benefits for pill taking, experiences during clinic visits. How might this change over time (e.g. with long-term use)?*

- 1. What do you think about the way the data from the monitor was used in clinic?

*Probe on counseling messages, interactions with the counselor, accuracy of data, time spent in clinic*

- 1. How was it having the monitor at home?
  2. We used the data from the monitor to send you SMS. How do you feel about those SMS?

*Probe on privacy, stigma*

- 1. Tell me how much value you think the monitor had for improving HIV care and treatment. How about the counselling? The SMS?

*Probe on this value in relation to other clinical programs or interventions (in the clinic or elsewhere in the health care system, real or theoretical)*

- 1. What recommendations do you have for using the monitor in future routine clinical care? Who should use it? How long should they use it?

*Probe on priorities for program implementation*

- 1. Is there anything else you would like to tell me about the monitor or any interventions that could be associated with it?

*Probe on the alarm*

- 1. Are there other ways in which you would prefer to see ART adherence supported?

*Remind participants that we are asking for their opinions but cannot necessarily influence the clinic itself.*

**READ:** Thank you for your participation. The interview is now over.

# Qualitative interview guide – Health care administrators/clinicians

**READ:** I would now like to get your reactions to the provisional results of this study.

1. Please tell me what you think of the monitoring device (now)? What do you like? What do you not like?
2. In what ways do you think the monitor was helpful for routine clinical care?

*Probe on individual benefits for patients, benefits for clinic flow and outcomes*

1. What are your concerns about the way the monitor was used for routine clinical care?

*Probe on individual benefits for pill taking, experiences during clinic visits. How might this change over time (e.g. with long-term use)?*

1. What do you think about the way the data from the monitor was used in clinic?

*Probe on counseling messages, interactions with the counselor, accuracy of data, time spent in clinic*

1. We used the data from the monitor to send SMS to clients taking ART. What do you think about the SMS?

*Probe on privacy, stigma, expense, staffing/logistics*

1. Do you think that there could be challenges with clinical implementation of the monitors and/or the associated interventions we used?

*Probe on any components of the intervention not addressed by the participant*

1. What sort of additional support might the clinic need to utilize the monitors and associated interventions in routine clinical care?
2. Tell me how much value you think the monitors had for improving HIV care and treatment. How about the counselling? The SMS?

*Probe on this value in relation to other clinical programs or interventions (in the clinic or elsewhere in the health care system, real or theoretical)*

1. What recommendations do you have for using the monitor in future routine clinical care? Who should use it? How long should they use it? What factors would influence your decision to invest or not invest in the monitors? How can you (in your current role) influence the decision to make this technology available to patients?

*Probe on priorities for program implementation*

*Probe on recommendations for device improvement*

1. What do you think of the ways we measured the value of the monitors? Would you recommend other methods?

*Refer the participant back to the report if needed*

1. Is there anything else you would like to tell me about the monitors or any interventions that could be associated with it?
2. Are there other ways in which you would prefer to see ART adherence managed in the clinic?

**READ:** Thank you for your participation. The interview is now over.
